# Help-Seeking Intention in Obsessive-Compulsive Disorder: Predictors and Barriers in South Africa

**DOI:** 10.3389/fpsyt.2021.733773

**Published:** 2021-09-24

**Authors:** Sarah Kate Hathorn, Christine Lochner, Dan J. Stein, Jason Bantjes

**Affiliations:** ^1^Department of Psychology, Stellenbosch University, Stellenbosch, South Africa; ^2^South African Medical Research Council Unit on Risk and Resilience in Mental Disorders, Department of Psychiatry, Stellenbosch University, Stellenbosch, South Africa; ^3^South African Medical Research Council Unit on Risk and Resilience in Mental Disorders, Department of Psychiatry and Neuroscience Institute, University of Cape Town, Cape Town, South Africa; ^4^Department of Global Health, Institute for Life Course Health Research, Stellenbosch University, Stellenbosch, South Africa

**Keywords:** obsessive-compulsive disorder, help-seeking, predictors, barriers, mental health service use, health belief model

## Abstract

**Introduction:** Many individuals with obsessive-compulsive disorder (OCD) delay seeking help, leading to greater illness severity, additional comorbidity, and increased functional impairment. Patterns of help-seeking for OCD have however not yet been described in South Africa, a low-and middle-income country with many health service challenges. Using the health belief model as a conceptual framework, study aims were to identify predictors of and barriers to help-seeking among South Africans with OCD.

**Methods:** Fifty adults with OCD completed an online survey to assess (1) socio-demographic characteristics, (2) OCD symptom severity, (3) treatment barriers, (4) perceived treatment benefits, (5) self-efficacy, and (6) help-seeking intention. Multiple linear regression analysis was used to establish predictors of help-seeking intention. Descriptive statistics were used to determine the most endorsed help-seeking barriers.

**Results:** 42.6% of the variance in help-seeking intention was explained by the investigated constructs (*R*^2^ = 0.426, *F* = 4.45 and *p* < 0.01). Perceived treatment benefits were the only significant predictor of help-seeking intention (*B* = 1.37, *t* = 5.16, and *p* < 0.01). More than a third (36%) of the sample endorsed wanting to handle the problem independently as a significant barrier, followed by treatment concerns (26%), affordability (22%), and shame (20%).

**Conclusion:** An innovative analysis of help-seeking patterns suggested that perceived treatment benefits were the only significant predictor of help-seeking intention among South African adults with OCD. Psychoeducation and mental health literacy programmes may be useful in increasing public appreciation of the benefits of OCD treatment, and in mitigating key help-seeking barriers.

## Introduction

Obsessive-compulsive disorder (OCD) is an underdiagnosed and undertreated psychiatric condition associated with clinically significant distress and functional impairment (1). OCD treatment has advanced significantly in the last three decades. Evidence-based pharmacological and psychotherapeutic treatments have been established (2). Clinical guidelines advise cognitive behavioral therapy (CBT) including exposure and response prevention (ERP) for mild functional impairment and selective serotonin reuptake inhibitors (SSRIs) and/or CBT for those with moderate functional impairment (3). At least half of patients who receive treatment will experience long-term remission as a result (4). A shorter duration of illness with early treatment consistently correlates with higher remission rates and more positive outcomes (4–6) whereas help-seeking delays are associated with greater illness severity, additional psychiatric comorbidity, and more significant functional impairment (7), illustrating the importance of early diagnosis and evidence-based intervention.

Despite these treatment advances, the majority of OCD patients do not reach out for professional help and remain untreated, or delay the help-seeking process for many years (2, 5, 7–11), contributing to what has been termed the “OCD help-seeking dilemma” [(6), pg. 8]. Even when individuals with OCD do seek help, the average period of delayed help-seeking is substantial, with estimates suggesting that it may take up to 11 years to seek professional help (7). Both structural and attitudinal factors may hinder seeking help for OCD [e.g., (7, 8, 12)]. The majority of studies have been conducted in high-income countries [e.g., (11–15)]. In low- and middle-income countries (LMICs) like South Africa, structural barriers may be particularly important. For example, in addition to psychological barriers such as stigma, feelings of shame, low self-efficacy, and poor mental health literacy as highlighted by international studies, challenges to seeking help for OCD may involve system-level obstacles in particular—including problems of affordability, transport, and geographical disparities in resources (16–18).

The health belief model (HBM) (18, 19) has been recommended as a valuable innovation in relation to understanding and addressing the problem of underutilization of mental health services (20–23). Drawing on the HBM as a conceptual framework, we aimed to (1) investigate the role of HBM constructs in predicting help-seeking intention among a group of South African adults with OCD, and (2), to determine the most endorsed barriers to help-seeking for OCD in this sample.

## Methods

This study employed a quantitative cross-sectional design, involving an online self-report survey with a battery of scales to measure the relevant HBM variables, administered to adult patients with a diagnosis of lifetime OCD. Data collection focused on *formal* help, which refers to assistance from mental health professionals qualified to provide assistance and treatment, including medical doctors, psychologists, psychiatrists, counselors, and other health practitioners (24). Although alternative, more informal sources of support exist and may play a role in OCD outcomes (25), formal help has garnered the most empirical support in the treatment of OCD (6, 10, 11, 26), and was therefore selected to be the focus of this study.

### Participants and Procedure

This study formed part of OCD research conducted at the Medical Research Council's (MRC) Unit on Risk and Resilience in Mental Disorders, in South Africa. Ninety-two participants (*n* = 92) were recruited from an existing database and received an email invitation from the principal investigator (Lochner) to participate. Those indicating interest (*n* = 54) were subsequently contacted with further study information and a link to the online survey. Inclusion criteria for the current study involved participants who were 18 years and older, living in South Africa, and who had obtained a lifetime diagnosis of OCD with/out comorbidity using the Structured Clinical Interview for DSM-5 (SCID-5) (27) within the last 3 years. This time frame was necessary in order to ensure the validity of the diagnosis. Exclusion criteria for the sample involved participants who were younger than 18 years of age, those who had not received a diagnosis of OCD in the last 3 years, or who were not living in South Africa.

### Instruments

Data collection was done using a comprehensive online self-report survey involving items to assess sociodemographic characteristics (gender, age, and level of education) and illness severity [i.e., the Florida obsessive-compulsive inventory (FOCI) severity scale]. The latter is a brief assessment measure of the severity of OCD symptoms (28) with good concurrent validity and strong internal consistency in other studies (28, 29), with Cronbach's alpha levels ranging from 0.89 to 0.92. In the current study, the Cronbach's alpha reliability coefficient was adequate (*a* = 0.82).

HBM-related constructs were measured as follows.

#### Treatment Benefits

Perceived treatment benefits were measured using the Attitudes Toward Seeking Professional Psychological Help Scale-Short Form (ATSPPH-SF) (27), which examines three core dimensions to assess perceptions of treatment benefits, namely “Openness to seeking professional help,” “Value in seeking professional help,” and “Preference to cope on one's own” [(28), p. 4], and has strong psychometric properties (30). In the current study, the Cronbach's alpha reliability coefficient was acceptable (*a* = 0.83).

#### Treatment Barriers

Treatment barriers were measured using the Barriers to Access Care Evaluation (BACE) scale, a comprehensive 30-item self-report instrument (31). Barriers include attitudes such as stigma and discrimination (i.e., “*concern that I might be seen as crazy*”), as well as structural barriers (i.e., “*not being able to afford the financial costs*”*)*, mental health literacy (i.e., “*wanting to solve the problem on my own*”), among others. It was not necessary to calculate the Cronbach's alpha reliability coefficient for the BACE, given that it is a formative construct—whereby the scale items inform the construct. However, the scale has demonstrated good psychometric properties in other studies (31).

#### Self-Efficacy

Self-efficacy [i.e., the belief in one's capacity to successfully accomplish a task or achieve a goal (32)] was measured using the general self-efficacy scale (GSES) (33), with items designed to assess self-efficacy, with a focus on positive self-beliefs and a sense of personal agency. The scale has strong internal consistency, with Cronbach's alpha reliability coefficients ranging between 0.76 and 0.90 across more than 20 studies, and has demonstrated good criterion-related validity (33). In the current study, the Cronbach's alpha reliability coefficient was acceptable (*a* = 0.89).

#### Help-Seeking Intention

Participants' help-seeking intention was rated on a scale from 1 (very unlikely) to 5 (very likely), with higher scores indicating increased likelihood of seeking help. Specifically, participants were asked, “How likely are you to seek help from a professional for OCD in the future?.” This one-item measure was based on a study conducted by Langley et al. (34), which utilized a similar help-seeking assessment method.

#### Treatment History

To enrich our understanding of formal help-seeking experiences within this sample, participants were asked to indicate whether they had received treatment for their OCD in the past (yes or no). Participants who indicated “yes” were presented with follow-up questions inquiring about the type of treatment sought, as well as the level of symptom change following treatment, measured using the Clinical Global Impression–Improvement scale (35).

### Data Analysis

**Figure 1:**
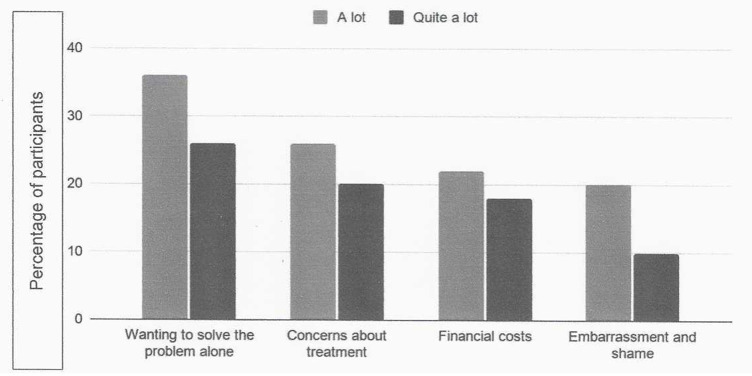
Top four barriers to help-seeking in the sample of individuals with OCD.

SPSS version 27 was used for data analysis. Descriptive statistics were calculated for sociodemographic characteristics, treatment history, predictor variables (i.e., symptom severity, perceived treatment benefits, treatment barriers, self-efficacy), and the outcome variable (i.e., help-seeking intention). In order to investigate whether HBM constructs significantly predict help-seeking intention in the sample, multiple linear regression analysis was performed. Regression assumptions of normality, homoscedasticity and multicollinearity were checked, and the significance level set at alpha = 0.05. A frequency table was generated to examine detailed responses regarding barriers to help-seeking, as well as a histogram to determine the top four barriers, in response to the second research aim (Figure 1).

### Ethical Considerations

The study protocol and participant information and consent documents were approved by the Institutional Review Board of Stellenbosch University, South Africa (S20/07/154). Informed consent was obtained from all participants after the nature of the procedures had been fully explained and before entering responses to the online survey. The consent form clearly stated that data would automatically anonymized. Participants were free to discontinue the survey at any point.

## Results

### Sample Characteristics

Fifty participants (*n* = 50, 29 [58%] female) with lifetime OCD took part. The mean age was 36.5 years, ranging from 19 to 59 years. The majority (58%) had university level education. The majority of participants (*n* = 46, 92%) had sought treatment in the past, and of these participants, 70% have received a combination of pharmacotherapy and psychotherapy. Scores on the Clinical Global Impression–Improvement scale indicated that 50% of participants reported much improved symptoms with any of the treatments received (Table 1).

**Table 1:** Treatment history.

	***n***	**(%)**	***f***
**Treatment sought**	50		
Yes		(92)	46
No		(8)	4
**Treatment type**	46		
Medication		(20)	9
Psychotherapy		(7)	3
Medication & psychotherapy		(70)	32
Other		(4)	2
**Symptom change with treatment**	46		
Very much improved		(15)	7
Much improved		(50)	23
Minimally improved		(26)	12
No change		(7)	3
Minimally worse		(0)	0
Much worse		(2)	1

### Predictors of Help-Seeking Intention

Results of the multiple linear regression are depicted in Table 2, and indicate that 42.6% of the variance in help-seeking intention is explained by the predictors in the model [*R*^2^ = 0.426, *F*_(7, 42)_ = 4.45 *p* < 0.01], with a moderate multiple regression coefficient (*R* = 0.65). Perceived treatment benefits was the only predictor variable that significantly predicted help-seeking intention [*B* = 1.37, *t*_(42)_ = 5.16, and *p* < 0.01].

**Table 2:** Regression results for the outcome variable: help-seeking intention^*^.

	**β**	**SEβ**	***B***	**SE** ***B***	***t***_**(42)**_	***p***
Intercept			−1.47	2.5	0.59	0.56
Age	−0.06	0.13	−0.01	0.01	−0.48	0.64
Gender	0.03	0.14	0.07	0.3	0.23	0.82
Education	0.12	0.15	0.15	0.18	0.82	0.42
Symptom severity	0.15	0.15	0.24	0.24	1	0.32
**Perceived treatment benefits**	**0.74**	**0.14**	**1.37**	**0.26**	**5.16**	**<0.01**
Self-efficacy	−0.14	0.14	−0.28	0.28	−0.99	0.33
Treatment barriers	0.2	0.15	0.41	0.31	1.34	0.19

* *Statistically significant (p < 0.01). Significant finding in bold type*.

### Barriers to Help-Seeking

Of the 30 barriers to help-seeking investigated here, the four most often endorsed were: wanting to handle the problem independently (endorsed by 36% of the sample), treatment concerns (26% of the sample), lack of finances (22% of the sample), and embarrassment and shame factors (20% of the sample) (Figure 1; Table 3).

**Table 3:** A description of the four most endorsed barriers to help-seeking in the study sample.

**Barrier**	**Not at all (%)**	**A little (%)**	**Quite a lot (%)**	**A lot (%)**
1) Wanting to solve the problem on my own	10	28	26	36
2) Feeling embarrassed or ashamed	32	28	10	20
3) Not being able to afford the financial costs involved	28	32	18	22
4) Concerns about the treatments available (e.g., medication side effects)	34	20	20	26

## Discussion

In our investigation with a cohort of adult South Africans with OCD, we found that perceived treatment benefits significantly predicted help-seeking intention, with the most frequently endorsed help-seeking barriers being (1) wanting to handle the problem independently, (2) treatment concerns, (3) lack of finances, and (4) embarrassment and shame factors.

Perceived treatment benefits were identified as a significant predictor of help-seeking intention in our study sample, echoing the results of international studies that have applied the HBM to mental health contexts [e.g., (30, 32)]. It has also been found to be a strong predictor of help-seeking intention for mental health concerns other than OCD, such as generalized anxiety disorder and general psychological distress, respectively (36). Both these prior studies (30, 32) were conducted in Australia, a high-income country where public access to psychological services is funded by the national health insurance scheme (34), with Australians readily having access to mental health care. This may be in contrast to the South African context where many people are uninsured and without access to psychological services (16). Perceived treatment benefits seem be linked to help-seeking intention across diverse settings and health systems however, irrespective of resource status. Notably, studies focusing on help-seeking for OCD specifically have also demonstrated similar results [e.g., (6, 37)], suggesting that positive beliefs about OCD treatment generally correlate with help-seeking processes. In these studies, positive beliefs about treatment were associated with having prior knowledge about OCD treatment, confidence in mental health practitioners, and information about psychological services through the media and by word-of-mouth (8, 38–40). Positive perceptions of treatment in this study may have been influenced by patients having treatment experiences in the past, which is supported by literature highlighting that prior help-seeking history significantly predicts future help-seeking intention for mental illness across cultural groups (41). In the current study, the treatment history data indicated that the majority of the sample had prior treatment, and approximately half of this group had improved.

Psychoeducation regarding the sources of help may encourage more positive beliefs about OCD treatment. Notably, a body of literature demonstrates that the public's general knowledge regarding OCD is generally poor (42–44). Rather than being recognized as a disabling mental disorder requiring psychiatric intervention, OCD is often trivialized in the lay media (45). The misperceptions associated with OCD in the public domain are problematic and contribute to individuals with OCD often being ill-equipped to recognize the nature of their symptoms and to seek appropriate help (43). Therefore, it can be suggested that educating the public about OCD and evidence-based treatment may encourage help-seeking and improve illness outcome. Similarly, South African research has emphasized the importance of attitudes, beliefs, and knowledge in relation to mental health service utilization (46). Thus, recommendations to improve mental health literacy relating to OCD in this setting may involve marketing and media campaigns that target widely held beliefs about OCD and advocate treatment benefits.

Our finding that symptom severity did not contribute to help-seeking intention is inconsistent with research suggesting that increased OCD symptom severity is associated with service use (9, 47–49). It is also contrary to the idea that individuals who are in greater distress or more functionally impaired are more likely to seek help. Our findings also suggested that sociodemographic characteristics were not statistically significant predictors of help-seeking intention—a finding that is inconsistent with some research highlighting a role for sociodemographic variables in mental health treatment utilization [e.g., (46)], with factors such as gender, race, gender, and sexual orientation significantly influencing treatment seeking. Similar to other mental health studies utilizing the HBM to predict mental health help-seeking [e.g., (30, 32)], our findings also suggested that self-efficacy did not contribute significantly to the variance in help-seeking intention.

The most endorsed help-seeking barrier in our sample involved the desire to solve the problem independently (“on my own”), mirroring an Australian study (13) where this also emerged as the most highly endorsed barrier to seeking help for OCD. Similarly, Goodwin et al. (50) found that a significant percentage of American adults with OCD (28.4%) endorsed a preference for self-reliance as a barrier to treatment-seeking. It is plausible that this finding relates to the strong degree of secrecy that is associated with OCD. Indeed, OCD has been termed “the secret illness” [(37), p. 7] and a large body of literature highlights the efforts undertaken by individuals to hide their OCD from friends, family, children, colleagues, and even therapists (8, 14, 38). Secrecy in OCD has also been linked to shame [e.g., (44)] and participants' preference for self-reliance might be explained by shame and embarrassment, which also emerged as an important barrier to help-seeking in this study. A preference for self-reliance has also emerged as a theme in studies focusing on other mental health problems in other settings (51–53). Further research is necessary to determine whether this barrier relates to OCD-specific features (such as secrecy and privacy) or reflective of more general mental health concerns. The second most endorsed help-seeking barrier in this study relates to concerns about the treatments that are available. Beliefs about treatment have an impact not only on help-seeking, but also on treatment adherence in the longer term, with implications for illness outcome. In many cases, fears about medication and its side-effects are fuelled by myths about the side-effects of psychiatric medications—involving sedative effects and personality changes—which are largely unfounded, particularly in relation to the newer SSRIs (54). Concerns about treatment may extend beyond medication and include reservations regarding psychotherapy for OCD, particularly ERP exposure exercises. Such exercises would involve exposing patients to obsessional triggers and cues so that they can learn to withhold compulsive rituals and break the obsessive-compulsive cycle (6). Many OCD patients spend much of their time avoiding obsessional cues, and therefore the prospect of undergoing an experience that actively induces obsessions would naturally be frightening. Avoidance behaviors, which are common among individuals with OCD, also often extend to treatment avoidance (55). This barrier reinforces the call for psychoeducation to address myths and quell fears surrounding psychological treatment and care. The third most endorsed help-seeking barrier in the current study relates to cost. Gold-standard guidelines for OCD treatment recommend that adults with mild to moderately impaired functioning should engage in more than 10 therapist hours of ERP therapy, with the option of a course of SSRI medication (3). Considering treatment costs in relation to the current average monthly salary of an adult in South Africa, psychotherapeutic treatment would be considered largely unaffordable. The final, fourth most endorsed barrier to help-seeking in the current study included embarrassment or shame, which may link to the burden of stigma surrounding OCD [e.g., (11, 13, 14, 37)]. The problem of stigma as a barrier to seeking help is not unique to OCD, but rather is a central theme in the experience of living with a mental health diagnosis as documented in other studies [e.g., (44, 45)]. Similarly, South African research has shown that stigma is a fundamental challenge in the delivery of mental health services (56), and although anti-stigma programs exist for mental health purposes in the country (56) there is a need to assess these interventions to better understand what is effective, and to identify where the gaps are. It is possible that feelings of shame and embarrassment may link to other factors, which are more specific to the experience of OCD. Notably, literature has highlighted that the *content* of obsessions (e.g., sexual/religious or *taboo* thoughts or images) may be associated with the endorsement of shame-related treatment barriers (8, 14). Psychoeducation would not only be helpful for patients to gain insight into their condition and encourage help-seeking, but also for members of the public to understand OCD better. This has the potential to minimize stereotypes and stigma surrounding the condition, which are processes that further fuel feelings of shame and embarrassment for patients with OCD.

Notably, the majority of endorsed help-seeking barriers in this study seemed to exist in the individual or psychological domain (i.e., a preference for self-reliance, treatment concerns, and shame factors) rather than on a structural level. For example, the majority of the sample (72%) did not endorse transport difficulties as a help-seeking barrier, in contrast to some international studies (14, 15). The few structural help-seeking barriers that were however reported in this study might be explained by the socio-economic status of the participants (not formally assessed), which—based on their education status—is likely higher than the wider community. This suggests that these participants may not have been as negatively affected by structural barriers.

This study is an important step in addressing the gap in the literature concerning help-seeking for OCD in South Africa. Future directions for research that have emerged include investigation of the viability of modified treatments, such as internet-based CBT (iCBT), in order to mitigate the identified barriers. This recommendation is based on research indicating that iCBT is cost-effective and accessible, and less likely to induce feelings of shame due to reduced therapist contact (57). Further qualitative inquiry could also bring about a deeper understanding of the challenges and barriers to help-seeking among persons with OCD.

Study limitations include the small sample size. Nevertheless, use of a purposive sampling technique, whereby participants were invited to take part based on the judgement of an expert (58), is a study strength, resulting in a sample that was relatively diverse in terms of their sociodemographic characteristics. Secondly, as most of these participants had sought treatment in the past (i.e., were not treatment naïve), the findings may not be generalizable to individuals with OCD in general. Furthermore, we did not have access to information confirming whether participants were receiving treatment at present, and it is possible that this may have influenced the study findings. The study was also limited by the self-report nature of the survey scales, which can be subject to bias. It is likely that there are other factors—not included in the HBM—that may also impact help-seeking intention and warrant further exploration. However, the HBM is one of the most established and comprehensive theories for explaining and predicting healthcare utilization, appropriate for the research aims given its sensitivity to both psychological and environment variables.

In conclusion, predictors of and barriers to help-seeking intention have not been well-studied. An innovative analysis of help-seeking patterns among South African adults with OCD suggested that perceived treatment benefits were the only significant predictor of help-seeking intention in this setting. Psychoeducation and mental health literacy programmes may be useful in increasing public appreciation of the benefits of OCD treatment, and in mitigating key help-seeking barriers. Further research into the subjective experiences of service use in South Africa with a larger study sample would add a deeper understanding of the challenges and experiences associated with OCD.

## Data Availability Statement

The original contributions presented in the study are included in the article/supplementary material, further inquiries can be directed to the corresponding author/s.

## Ethics Statement

This study involved human participants and was reviewed and approved by Health Research Ethics Committee, Faculty of Medicine and Health Sciences, Stellenbosch University, South Africa. The participants provided their written informed consent to participate in this study.

## Author Contributions

CL and SH were instrumental in the acquisition, analysis, and interpretation of data. SH made the first draft and all other authors revised it critically. All authors made substantial contributions to the conception or design of the work, provided approval for publication of the content, and agree to be accountable for all aspects of the work.

## Funding

The financial assistance of the National Research Foundation (NRF) of South Africa (reference number: BRIC190221420217; awarded to CL) and the South African Medical Research Council (Career Development Grant; awarded to JB) towards this research is hereby acknowledged.

## Author Disclaimer

Opinions expressed and conclusions arrived at, are those of the author and are not necessarily to be attributed to the NRF.

## Conflict of Interest

The authors declare that the research was conducted in the absence of any commercial or financial relationships that could be construed as a potential conflict of interest.

## Publisher's Note

All claims expressed in this article are solely those of the authors and do not necessarily represent those of their affiliated organizations, or those of the publisher, the editors and the reviewers. Any product that may be evaluated in this article, or claim that may be made by its manufacturer, is not guaranteed or endorsed by the publisher.
